# Brucellosis remains a neglected disease in the developing world: a call for interdisciplinary action

**DOI:** 10.1186/s12889-017-5016-y

**Published:** 2018-01-11

**Authors:** K. A. Franc, R. C. Krecek, B. N. Häsler, A. M. Arenas-Gamboa

**Affiliations:** 1 The University of Georgia, College of Veterinary Medicine, Athens, GA 30602 USA; 2 Global One Health, Office of the Dean, College of Veterinary Medicine & Biomedical Sciences, Texas A&M Veterinary Medical Center, Texas A&M University, 4461 TAMU, College Station, TX 77843-4461 USA; 3 Department of Veterinary Pathobiology, College of Veterinary Medicine & Biomedical Sciences, Texas A&M University, 4467 TAMU, College Station, TX 77843-4467 USA; 4 Department of Zoology, University of Johannesburg, P.O. Box 524, Auckland Park, Johannesburg 2006 South Africa; 5 Royal Veterinary College, Hawkshead Lane, North Mymms, Hatfield, AL9 7TA UK

**Keywords:** Brucellosis, *Brucella*, One health, Zoonosis, Infectious disease, Global health (10 maximum)

## Abstract

**Background:**

Brucellosis is an endemic zoonotic disease in most of the developing world that causes devastating losses to the livestock industry and small-scale livestock holders. Infected animals exhibit clinical signs that are of economic significance to stakeholders and include reduced fertility, abortion, poor weight gain, lost draught power, and a substantial decline in milk production. In humans, brucellosis typically manifests as a variety of non-specific clinical signs. Chronicity and recurring febrile conditions, as well as devastating complications in pregnant women are common sequelae.

**Discussion:**

In regions where the disease is endemic, brucellosis has far-reaching and deleterious effects on humans and animals alike. Deeply entrenched social misconceptions and fear of government intervention contribute to this disease continuing to smolder unchecked in most of the developing world, thereby limiting economic growth and inhibiting access to international markets. The losses in livestock productivity compromise food security and lead to shifts in the cognitive competency of the working generation, influence the propagation of gender inequality, and cause profound emotional suffering in farmers whose herds are affected. The acute and chronic symptoms of the disease in humans can result in a significant loss of workdays and a decline in the socioeconomic status of infected persons and their families from the associated loss of income. The burden of the disease to society includes significant human healthcare costs for diagnosis and treatment, and non-healthcare costs such as public education efforts to reduce disease transmission.

**Conclusion:**

Brucellosis places significant burdens on the human healthcare system and limits the economic growth of individuals, communities, and nations where such development is especially important to diminish the prevalence of poverty. The implementation of public policy focused on mitigating the socioeconomic effects of brucellosis in human and animal populations is desperately needed. When developing a plan to mitigate the associated consequences, it is vital to consider both the abstract and quantifiable effects. This requires an interdisciplinary and collaborative, or One Health, approach that consists of public education, the development of an infrastructure for disease surveillance and reporting in both veterinary and medical fields, and campaigns for control in livestock and wildlife species.

## Background

Brucellosis is a zoonotic disease that severely hinders livestock productivity and human health worldwide. The burden that the disease places specifically on low-income countries has led the World Health Organization (WHO) to classify it as one of the world’s leading ‘neglected zoonotic diseases’ [1]. Brucellosis is caused by bacteria of the genus *Brucella*, with *Brucella abortus*, *Brucella melitensis,* and *Brucella suis;* infecting cattle, small ruminants, and swine, respectively, being species of particular importance in human and livestock infections worldwide. Other species of concern include *Brucella canis,* infecting dogs, and *Brucella ovis*, infecting sheep [2].

In animals, brucellosis is highly contagious and cross-species transmission of certain *Brucella* spp*.* can occur [2]. Mucosal contact with aborted fetuses and fetal membranes, which contain large amounts of the bacteria, is an important means of transmission in livestock [3]. Infected livestock exhibit clinical signs of great economic significance to stakeholders (i.e., small scale livestock farmers, meat and milk industry, human communities, etc.), including reduced fertility, abortion, and a substantial decline in milk production over an animal’s lifespan [4]. In humans, brucellosis typically manifests as a range of non-specific clinical signs including malaise, fatigue, arthritis, and fever. Chronicity and recurring febrile conditions with joint pain are common sequelae [5]. The acute and chronic symptoms of the disease can result in a significant loss of work days and consequential disparity in the socioeconomic status of infected persons and their families. Human-to-human transmission can occur transplacentally, via breastfeeding, and rarely through sexual intercourse, organ transplantation and blood transfusions [6]. Transmission can also occur through direct contact with infected animals, their tissues (e.g. placenta or aborted tissues), or their products (e.g. dairy) [5]. While pasteurizing milk is an effective means to kill *Brucella* and prevent infection in humans, this precaution is not routinely practiced in some resource limited communities because of long- standing cultural practices and a generalized lack of understanding by the public about the dangers of consuming raw milk [7]. In Egypt, for example, social misconceptions about the nutritive quality of pasteurized milk has led to a reduction in consumption of commercial milk products, leaving raw milk from small-scale dairy producers to account for up to 80% of the total milk industry (up to 4 billion liters per year), according to the chairman of the Egyptian Chamber of Food Industries [8]. Exacerbating the problem are the food products from informal markets that predominate in low and middle income countries and often escape meeting the appropriate health and safety standards before reaching the consumer [9].

In low and middle-income countries, misconceptions about *Brucella’s* true incidence often arise from underreporting and insufficient monitoring data, a lack of financial resources and capacity, and efforts between veterinarians and human medics. All contribute to the continued prevalence of brucellosis [10]. Paired with the presence of rampant social stigmas and mistrust regarding government intervention, this has allowed *Brucella* to increase unchallenged in the marginalized human and animal populations of the world. For example, in India, religious beliefs denounce practices that include the test and slaughter of livestock for infectious diseases, and small scale livestock keepers have been known to avoid diagnostics for fear of consequences (e.g., slaughter of their entire herd) if a single animal were to test positive for brucellosis [9, 11]. Zinsstag et al., 2006 [12] discusses nomadic pastoralists who live in many areas of the world and their vulnerability to brucellosis because of close contact during animal husbandry and consumption of livestock products. He further discusses that though they are socially marginalized, they make significant contributions to national gross domestic products (GDPs) by making marginal lands more productive.

This paper aims to synthesize both the well and poorly understood factors that account for the wide-ranging socioeconomic impact of brucellosis in regions of the world where it is endemic. We address dimensions of the *Brucella* discussion that have to date been neglected, largely because their abstract nature makes them difficult to assess quantitatively. We also aimed at assisting researchers in their prioritization and decision-making processes by comprehensively assessing the complexities associated with reducing the global impact of brucellosis. To accomplish this, we used economic frameworks, published by Rushton et al. 1999 [13], and Jo in 2014 [14], to explore the impact that brucellosis has in regions of the world where it currently exists. Following this, we offer an assessment of the collaboration required to address this issue, along with a call for action; that is developing a One Health approach through the collaboration of human, animal and public healths to demonstrate an added value of cost savings and/or improved health by working in concert rather than alone.

## Discussion

### Worldwide distribution of brucellosis

We use published case studies and reviews to highlight the estimated prevalence of brucellosis in regions where it is endemic. However, it should be noted that because many areas with endemic brucellosis have poor medical and veterinary infrastructures, the true incidence of disease is likely to be markedly under-reported [15].

### Middle East

A review paper published by Musallam et al., 2015 [16] compiled the available prevalence data sets for the region of the Middle East and screened them for quality of reporting. The screening process included reviewing retrieved abstracts by the primary author based on the following inclusion of four criteria. First, the research was original and studied a human or animal population in a Middle Eastern country; second, the article was published in a peer-reviewed section of a journal; and, third, that the research was written in Arabic, English, French or Persian. The fourth criterion was to determine if the research provided estimates of the frequency of *Brucella* spp. infection in domestic ruminants and/or humans, or estimates of the strength of the association between *Brucella* spp. (sero)-positivity in humans and potential risk factors. If required for clarification, the full text was retrieved and the article subjected to quality assessment and data extraction. Per their data, *B. melitensis* and *B. abortus* have been confirmed in most countries of the Middle East [16]. The seroprevalence results from small ruminant populations in these regions is remarkable. For example, studies from Egypt in 2008 suggest a brucellosis true seroprevalence rate of: 12.2% prevalence in sheep, 11.3% prevalence in goats, 41.3% true prevalence of sheep and 32.2% of goats in infected villages [17]. Published data from other countries in the Middle East reinforce that brucellosis is a significant problem in the region. In Saudi Arabia, for example, consumption of raw milk by people resulted in a 3.0 to 3.8 times greater probability that an individual would be seropositive for *Brucella* antibodies on serology [18]. This is consistent with reports from Jordan, where sheep seroprevalence of brucellosis was estimated to be 2.2% at the individual animal level and 45% at the herd level [19].

### Africa and Asia

In 2013, a study by McDermott et al. [4] was conducted to estimate the economic impact of brucellosis in the developing nations of Africa and South/ Southeast Asia. In all, 259 studies on brucellosis from these regions were analyzed and encompassed observations from 500,000 animals, 30,000 people, and 600 food samples. While the sensitivity and specificity of surveillance and diagnostic tests can over- or underestimate the true prevalence of disease, the compiled information from this work provides a valuable estimate. The data revealed an average prevalence range of 0–88.8% in sheep and goats, 0–68.8% in cattle, 0.4–20% in camels, and 0–12.9% in other species (pigs and dogs). In high risk human populations, such as veterinarians, livestock handlers, and abattoir workers, the average prevalence was 11%. Among hospital patients in which brucellosis was consistent with the clinical picture, the prevalence was 7% [4]. These data suggest that brucellosis is endemic across the African and Asian continents and is a major cause of disease in humans and livestock [4].

### Central and South America

A retrospective analysis of brucellosis in Latin America was published by Lucero et al. in 2008 [20]. Overall, the study reports that in Latin American countries, the prevalence of brucellosis in cattle ranges from 0.5% - 10%. However, the prevalence of the disease in suids is currently unknown. There is also a lack of significant data about *Brucella* in small ruminants from the region. In Argentina, official data reports note that the estimated prevalence of brucellosis in cattle is 10–15% among herds and 4–5% among individual animals, the species also composing the largest sector of the livestock industry [20]. In Central America, *B. abortus* and *B. suis* have been identified in every country and *B. melitensis* in Guatemala, with the prevalence rate of brucellosis in Central American cattle estimated to be between 4 and 8% [21].

### Overview of brucellosis impact in livestock where it is endemic

**Fig. 1:**
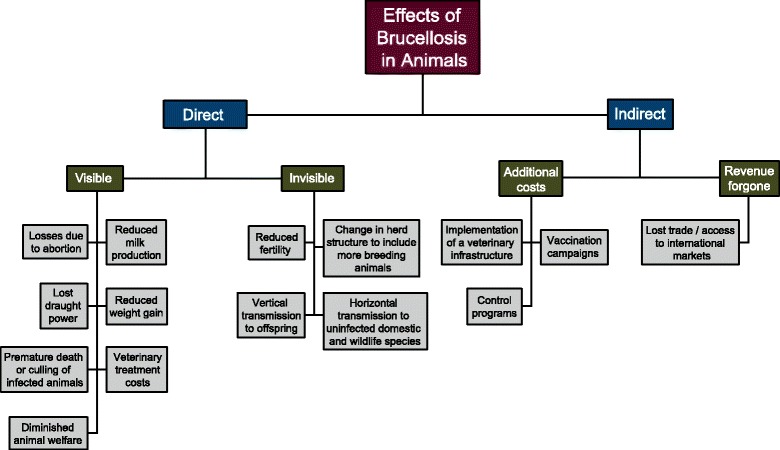
Framework to assess the effects of livestock brucellosis in regions where the disease is endemic, adapted from Rushton et al., 1999

In this section, we adapt the framework proposed by Rushton et al. 1999 [13] to assess the impact of brucellosis infection in livestock in regions where it is endemic. This framework is outlined in Fig. 1. It consists of determining the direct and indirect economic losses attributable to brucellosis and highlights the wide breadth of consequences that brucellosis has on the livelihood of livestock stakeholders in low-resource communities. Direct effects include those that are visible, or directly evident to the stakeholder, and those that are invisible, or that include forgone production potential [13]. In the case of brucellosis, visible losses include livestock abortion, reduced milk production, lost draught power, reduced weight gain from chronic infections and ill-thrift, premature death or culling of unproductive stock, veterinary costs associated with treating clinically ill animals and diminished animal welfare. In endemic areas, *Brucella* spp*.* can cause a significant reduction in herd productivity that compromises food security and the livelihood of farmers who depend on the sale or trade of surplus meat, dairy, and offspring from their animals. For example, a 6-year brucellosis control plan implemented on a Mexican dairy farm with 300 cows revealed an increase in average milk production by 6 L per day by the conclusion of the study [22]. Multiple studies have also found a correlation between seropositivity for *Brucella* spp*.* and history of abortion [4]. In Ethiopia, seropositive livestock were found to be 4.7 (cattle) and 6.9 (goats) times more likely to have aborted in their lifetime when compared to seronegative animals [23]. This demonstrates that while the decreases in livestock productivity caused by brucellosis induced abortion and reduced milk production are multifactorial, severe production losses can occur as a result. In 2002, the Food and Agriculture Organization (FAO) released a study that estimated the effects of eliminating bovine brucellosis in sub-Saharan Africa. The modelling estimates predicted that eradicating the disease from this region would generate between USD $0.497– USD $1 billion in additional income potential for stakeholders on an annual basis [24]. Furthermore, a study conducted in India in 2015 attributed a median loss of USD $3.4 billion of revenue to the livestock sector from the production losses, reduction in fecundity, and premature deaths of animals infected with brucellosis [25]. Additional visible losses involve the negative effects of the disease specifically in draught animals. Carpal hygromas are common clinical signs of chronic brucellosis in cattle [26] and can result in joint pain, inflammation, and a reduction in mobility that can be particularly disabling for them. This has the potential to severely limit their usefulness as a means of transportation or draught power and can compromise the income of farmers or community members who depend on them for these purposes.

The direct but invisible losses include reduced herd fertility and the costs associated with changing the herd structure to compensate for the overall reduction in productivity and fertility (Fig. 1). In cattle, infertility due to brucellosis is caused by post-abortion metritis and retained placentas [27]. Additional invisible losses are associated with the transmission of *Brucella* spp. from infected livestock to wildlife or feral animals that can then transmit the disease to surrounding herds. Brucellosis has been successfully eradicated in several high- income countries through the implementation of multi-faceted control strategies that include vaccination and test-and-slaughter of infected livestock [28, 29]. The World Organisation for Animal Health (OIE) declares that several countries in Western and Northern Europe, Canada, Japan, Australia, and New Zealand are free of *Brucella*. However, eradication of brucellosis proves difficult even for developed countries, such as the United States, because of the ability of some *Brucella* spp*.* to infect multiple species and to reside in feral populations of ruminants and swine [30, 31]. Once a reservoir host for brucellosis becomes established in a region due to this type of transmission event, it is much more difficult to eradicate and requires a substantial increase in resources for effective control [32]. For example, in the United States, USD $3.5 billion was invested in a brucellosis eradication program that successfully decreased the prevalence of bovine brucellosis from 11.5% to 0.0001%. However, despite this investment, elk and bison in the Greater Yellowstone Ecosystem of Wyoming, Idaho, and Montana remain reservoirs for the disease – signifying the difficulties of eradication when wildlife reservoirs for *Brucella* spp. exist. Because nomadic styles of livestock rearing are common in the developing world, the transmission of brucellosis from infected to naïve herds of livestock via feral animals or wildlife species that act as reservoirs is a major concern. A study conducted in 2015 in the Katavi-Rukwa ecosystem of Tanzania detected anti-*Brucella* antibodies in humans, cattle, goats, and buffalo residing in the region. This is significant because farmers in this area practice agro- pastoral livestock rearing and the co-mingling of wildlife and domestic species is common. In other words, it suggests that brucellosis has cyclic transmission capabilities that involve humans, livestock, and wildlife species who live in close proximity to each other [33].

Apart from losses due to disease, which are one source of economic cost, brucellosis also causes indirect financial expenditures for disease management and forgone revenue when a disease is present in a region. Costs for veterinary services, vaccination, diagnostics, farmer indemnification, and a system for animal identification and maintenance of surveillance records are incurred when a disease management plan is implemented on a regional or national level. However, before a control plan can be pursued, a stable veterinary infrastructure and system for animal identification must be in place. While the resources allocated to developing this infrastructure can be greater than the capital of product preserved after an effective *Brucella* control plan is established, it is important to note that this is essential for decreasing the incidence of all livestock and zoonotic diseases. Therefore, this cost can be distributed and analyzed alongside all diseases that affect livestock productivity in a region of interest.

Furthermore, forgone revenue related to brucellosis includes trade restrictions from areas endemic with *B. melitensis, B. abortus,* and *B. suis*. These *Brucella* spp*.* are listed as notifiable diseases by the OIE – a list made in compliance with the Sanitary and Phytosanitary Agreement of the World Trade Organization that helps to guide policy development concerning the international trade of products contaminated with specific biological agents [34].

### Overview of brucellosis impact in humans where it is endemic

**Fig. 2:**
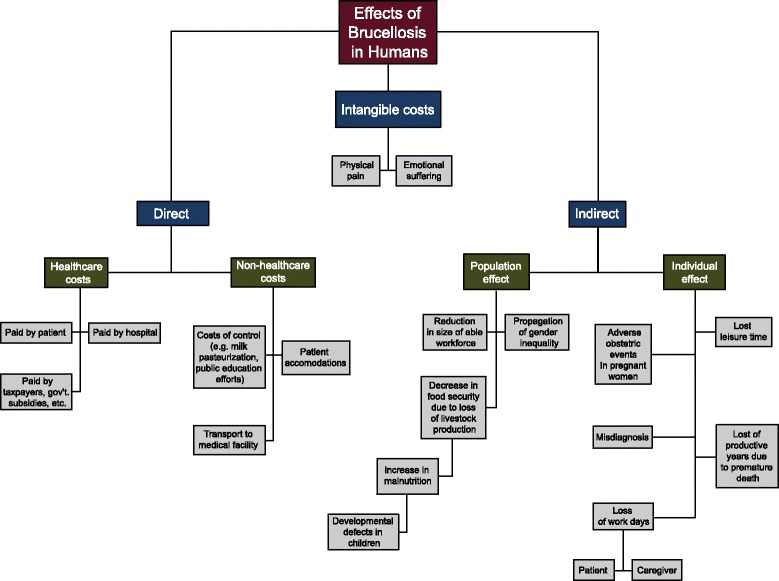
Framework to assess the effects of human brucellosis in regions where the disease is endemic, adapted from Jo, 2014

We used the framework proposed by Jo (2014) [14] to assess the impact of endemic brucellosis in human populations. A thorough compilation of the intangible, direct and indirect costs as presented in the literature is discussed as they relate to the cost of illness (COI), or burden of disease, when brucellosis is present in a population (Fig. 2). According to the framework, intangible costs include those that diminish a patient’s quality of life but that cannot be easily standardized between individuals and therefore typically lack a predictable monetary value. Direct costs include healthcare costs, or medical expenditures for the diagnosis, treatment and management of clinically-ill patients, and non-healthcare costs, or those that provide a patient with access to care [14]. Indirect costs include those associated with the morbidity and mortality of a disease that specifically affect the patient and society in which the patient lives [14].

In regions where brucellosis is endemic, intangible costs contributing to the overall burden of disease include physical pain and emotional suffering. In about half of all cases, the disease presents as an acute febrile condition with patients commonly reporting joint and back pain, fatigue, headaches, and a loss of appetite [35]. However, infection can cause complications in any organ system, and a delayed onset of symptoms and chronic form of the disease are common [35]. Emotional suffering results directly from the decrease in quality of life of persons experiencing clinical symptoms and from the effects that the disease has on the livelihood of small scale livestock stakeholders. In general, farmers with small holdings of livestock, the typical production scheme in low-income regions, suffer from increased stress and a lack of access to support or mental health services [36]. In 2016, a study conducted in India revealed that farmers were more likely to commit suicide because of socioeconomic factors like indebtedness rather than mental disease [37]. Extrapolate these findings to a disease like brucellosis, which has considerable negative implications on livestock productivity, and it is evident that farmers’ feelings of grief, hopelessness, depression, and suicide are likely amplified in regions where the disease is perpetuated.

Direct effects of brucellosis in human populations include the associated healthcare and non-healthcare costs of the disease. Healthcare costs include the expenses related to laboratory diagnostics, physician services, hospitalization, treatment, and medications for the clinically-ill.

Depending on the existing healthcare system where the patient lives, the responsibility for paying these costs can fall on the patient, patient’s family, insurer, hospital, taxpayers, or government. Human brucellosis is a disease that results in significantly higher healthcare costs compared to the average patient seeking medical care [38]. A study conducted in southern Israel revealed that healthcare utilization costs for brucellosis patients was USD $57 (*p < 0.05*) greater before diagnosis and USD $947 (*p < 0.001*) greater one year post diagnosis when compared to non-brucellosis cases that were matched by age, sex, hospital, and primary care physician [38]. The higher costs of patients post diagnosis stem from higher utilization of medications, spinal/skeletal diagnostic procedures, emergency room visits, and laboratory tests [38].

Non-healthcare costs include expenses related to a patient’s access to care and the prevention of disease. Transport to and from medical facilities, housing accommodations while care is being received, and a loss of work days or leisure time when seeking medical attention can amount to significant expenses that the patient or their families are responsible for paying out-of-pocket. It should also be noted that while individuals in low-resource settings have an increased prevalence of health complications and shorter life expectancy, access to health services in these areas is significantly lacking [39]. Thus, it is evident that the costs for acquiring medical care are amplified for persons affected by brucellosis because it is a disease predominantly affecting the poor. Preventative measures to control brucellosis in the human population are other important non-healthcare costs to consider. Risk factors for *Brucella* infection include occupations that involve contact with livestock or their products and the human consumption of raw milk products such as ghee and un-boiled milk [40]. The risk factors of brucellosis may vary from country to country and region to region, but most risk factors are similar. Consumption of unpasteurized milk and milk products plays a very important role in the transmission of this infection from animals to humans, in addition to direct contact with infected animals and their secretions. The best way to control this ubiquitous infection is through the One Health approach which involves human health, animal health, and environmental health [41]. The dissemination of educational information about disease prevention to regions where *Brucella* is endemic is therefore essential for controlling the disease [40]. Furthermore, because infected animals and their products are the primary source of human infections, control of brucellosis in livestock and the development of an effective veterinary infrastructure is an important step to mitigating the disease in humans [42].

In regions where brucellosis is endemic, indirect effects include adverse obstetric events in pregnant women, delays in treatment due to misdiagnosis, lost leisure time and productive years due to illness or premature death, and a loss of work days for both the patient and caregiver. Furthermore, misdiagnosis is common when patients present with brucellosis because the symptoms can be vague, non-specific and mimic other diseases that persist in low-resource settings such as malaria and typhoid [43]. This misdiagnosis often leads to a delay in treatment and can result in long term complications from the disease [44], as well as create discrepancies between the reported and actual number of human cases in a region. Pregnant women infected with brucellosis can develop devastating complications such as preterm labor and delivery, congenital malformations, low birth weight, abortion, and fetal, neonatal and maternal death [45]. The severity of clinical signs if a woman becomes infected does not correlate to the likelihood that she will suffer a miscarriage [46]. In other words, she may miscarry or suffer other severe complications without ever recognizing a need to seek treatment until after it is too late for medical intervention to be effective.

The population effect of brucellosis in regions where the disease is endemic includes a decrease in food security, a reduction in the size of the workforce which is physically able, and the propagation of gender inequality. In these settings, the reduction in milk production and fertility caused by *Brucella* infections in livestock drastically compromises food security and increases malnutrition in already severely nutrition poor communities. For example, in 2002 the FAO estimated that an average of 371,000 tons of additional meat and 616,000 tons of additional milk would have been generated during that year if brucellosis was eradicated in sub-Saharan Africa alone [24]. This is particularly important because it is well accepted that adequate nutrition is essential in early childhood for cognitive development and that the propagation of malnutrition in a population can have serious consequences for society as a whole [47]. It is also important to note that in some cases, increased livestock production allows women to increase their control over personal income and assets, which is an important mechanism to reduce gender inequalities in patriarchal societies [48]. Controlling brucellosis in livestock populations increases production and in turn aids women, the primary livestock caretakers in most low-income communities [48], to overcome social oppression and dependency.

### Interdisciplinary call to action

One of the most effective means to reduce the burden of any disease is to reduce its prevalence in regions where it is endemic. Vaccine campaigns, community outreach and education, and conducting accurate disease surveillance estimates are the cornerstones of these efforts. However, there are currently no *Brucella* vaccines available for use in humans that are considered to be safe and effective [49]. Furthermore, the vaccines available for use in animals do not allow for differentiation between naturally infected and vaccinated animals on serological diagnostic tests [50], a distinction that is imperative for assessing the success of any vaccination program and for estimating true disease prevalence after vaccines have been administered in a respective region. Hence, research funding that is focused on developing improved vaccines and diagnostic tools for brucellosis is an essential first step of the ever-evolving strategy to solve the *Brucella* puzzle.

The implications of brucellosis that are synthesized in this paper suggest that to reduce or eradicate it, an interdisciplinary, or “Global One Health”, approach is essential. One Health encompasses the methodology that human, animal and environmental health are closely intertwined, and that improvement in one of these areas is contingent on the interdependence of all three. Thus, collaboration between professionals across multiple disciplines and sectors is imperative to reaching solutions that lead to the mitigation of infectious diseases such as brucellosis ultimately adding economic value. In 2003, an economic model for brucellosis was simulated in Mongolia to assess the success and financial return of livestock vaccination on the incidence of the disease in humans. The study demonstrated that an investment of USD $8.3 million for mass vaccination of cattle and small ruminants using commercially available vaccines would result in a USD $26.6 million return (or net present value of USD $18.3 million).

This is an example of increased livestock production and profitability, while at the same time reducing human healthcare costs, income loss, and coping costs in the target region [51]. This demonstrates the importance of implementing a holistic and multi-disciplinary approach to the mitigation of brucellosis by incorporating strategies that break the cycle of transmission from animals to humans and can be applied to other zoonotic diseases. Similarly, in 2009, the United States Agency for International Development (USAID) created an initiative called PREDICT. PREDICT successfully began a movement of collaboration between government ministries, scientific institutions, local organizations and stakeholders to promote human and animal health, effective resource management, and economic development in twenty countries [52]. With the success of the Mongolia and PREDICT models in mind, and considering the unique pathology of brucellosis and its far-reaching socioeconomic effects in the resource-poor countries of the world where livestock of small holder farmers is their livelihood and “bank account,” it is evident that partnerships between veterinary, medical, environmental, economic, cultural, policy, and societal experts are essential to reducing the burden of this disease across the globe.

## Conclusions

Brucellosis is an endemic zoonotic disease in low, middle, and high-income countries that causes devastating losses to the livestock industry including small-scale livestock holders. It places significant burdens on human healthcare systems and limits the economic potential of individuals, communities, and nations where such development is especially important to diminish the prevalence of poverty. The implementation of public policy focused on mitigating the socioeconomic effects of brucellosis in human and animal populations is desperately needed. The interdisciplinary “One Health” nature of the effects that brucellosis has indicate that collaboration of veterinary, medical, public health, cultural, economic and social experts is needed to effect a change in disease burden.

Important topics for future studies include generating assessments about the impact of brucellosis as it relates to the exponential growth of the human population and the increasing intensification of livestock production systems worldwide. This, coupled with projections that address the cost-effectiveness of mitigation measures, will be vitally important for policy-makers to make informed decisions to manage this disease. While there are still many questions to be answered regarding the true burden of brucellosis worldwide, control and mitigation in regions where it is endemic will help to improve food security, household income, and human and animal health in low-resource communities. Allocating resources towards the research and development of improved *Brucella* vaccines and diagnostic tools is an important facet of addressing the problem. It is essential to engage community leaders in developing tools and resources which are effective in building outreach and education campaigns at the local level. It is especially critical to apply initiatives that teach people how to protect themselves, their families, and their communities from the disease through sustainable improvements and education. Some possible focus areas are sanitation and hygiene, food safety, biosecurity, and livestock management techniques that are culturally and financially feasible given the location of interest. This approach will inevitably lead to healthier populations of humans and animals at the community level and result in a ripple effect that builds more stable economies and improves the health and productivity of multiple species on a global scale.

## Abbreviations

FAO: Food and Agriculture Organization
Health COI: Cost of Illness
OIE: World Organisation for Animal Health
USAID: United States Agency for International Development
USD: United States Dollar
WHO: World Health Organization
